# Prognostic value of platelet indices in patients with acute pulmonary thromboembolism

**DOI:** 10.34172/jcvtr.2020.09

**Published:** 2020-02-12

**Authors:** Samad Ghaffari, Nashmil Parvizian, Leili Pourafkari, Ahmad Separham, Reza Hajizadeh, Nader D Nader, Elnaz Javanshir, Nariman Sepehrvand, Arezou Tajlil, Babak Nasiri

**Affiliations:** ^1^Cardiovascular Research Center, Tabriz University of Medical Sciences, Tabriz, Iran; ^2^Department of Anesthesiology, State University of New York at Buffalo, Buffalo, NY, USA; ^3^Department of Cardiology, Urmia University of Medical Sciences, Urmia, Iran; ^4^Mazankowski Alberta Heart Institute, University of Alberta, Edmonton, Alberta, Canada

**Keywords:** Pulmonary Thromboembolism, Platelet, Mean Platelet Volume, Platelet Distribution Width, Mortaity

## Abstract

***Introduction:*** Given the role of platelets in thrombus formation, markers of platelet activation may be able to predict outcomes in patients with acute pulmonary thromboembolism (PTE).

***Methods:*** In a prospective cohort study, 492 patients with acute PTE were enrolled. Patients were evaluated for platelet indices including mean platelet volume (MPV), platelet distribution width (PDW), and platelet-lymphocyte-ratio (PLR), as well as for the simplified Pulmonary Embolism Severity Index (PESI) risk score. The primary endpoint was in-hospital all-cause mortality. Major adverse cardiopulmonary events (MACPE, composite of mortality, thrombolysis, mechanical ventilation and surgical embolectomy during index hospitalization) and all-cause death during follow-up were secondary endpoints.

*** Results:*** MPV, PDW and PLR were 9.9±1.0 fl, 13.5±6.1%, and 14.7±14.5, respectively, in the total cohort. Whilst MPV was higher in those with adverse events (10.1±1.0 vs 9.9±1.0 fl; *P*= 0.019), PDW and PLR were not different between two groups. MPV with a cut-off point of 9.85 fl had a sensitivity of 81% and a specificity of 50% in predicting in-hospital mortality, but it had lower performance in predicting MACPE (Area under the curve: AUC 0.58; 95%CI 0.52-0.63) or long-term mortality (AUC 0.54; 95% CI 0.47-0.61). The AUC for all these three markers were lower than the AUC calculated for the simplified PESI score (0.80; 0.71-0.88).

***Conclusion:*** Platelet indices had only fair-to-good predictive performance in predicting in-hospital all-cause death. Established PTE risk scoring models such as simplified PESI outperform these indices in predicting adverse outcomes.

## Introduction

Pulmonary thromboembolism (PTE) is a major cause of cardiovascular mortality worldwide with a case fatality rate of 15% within the early post-diagnosis phase.^1^ It incurs 7-10 billion dollars annually to the healthcare system only in the United States.^2^ Risk stratification and prognostication is of great importance in patients who present to emergency department with acute PTE, and follows two main purposes: Identifying low-risk patients that can be treated as an outpatient and finding high-risk cases that may benefit from second-line therapies such as thrombolysis or surgical embolectomy. Beside risk stratification tools such as Pulmonary Embolism Severity Index (PESI),^3,4^ readily available blood parameters have been recently proposed to be predictors of clinical outcome in venous thromboembolism.

Given the established role of platelets in thrombogenesis, platelet activation is considered as an expected phenomenon in PTE. Mean platelet volume (MPV) and platelet distribution width (PDW) are markers of platelet production and activation. Higher MPV (i.e. larger platelets) is shown to be associated with the presence of more granules, higher levels of thromboxane A2, rapid aggregation with collagen, and more glycoprotein Ib and IIb/IIIa receptors.^5,6^

Furthermore, the Leukocytes’ physiologic response to stress usually presents with an increase in the sympathetic activity and cortisol levels as well as an increase in the number of neutrophils which is associated with a decrease in the number of lymphocytes. This leads to the extravasation of neutrophils into the affected area.^7^ Platelet-lymphocyte ratio (PLR), as a surrogate marker of inflammation, may be used to explore the link between thrombosis and inflammation in PTE, which is mediated by the production of reactive oxygen species and higher levels of myeloperoxidase enzyme,^8^ the consequent oxidative stress,^9^ and the release of pro-inflammatory cytokines such as IL-6 and TNF-α.^10,11^

In this study, we measured the levels of MPV, PLR and PDW in a cohort of patients who were diagnosed with acute PTE in emergency department to investigate the predictive performance of these biomarkers in predicting adverse outcomes in this patient population.

## Materials and Methods

In a single-center prospective cohort study, 492 patients with a confirmed diagnosis of acute pulmonary embolism were enrolled at Madani Heart Hospital in Tabriz, Iran, between March 2011 and March 2015.

### Study population

Patients with an age older than 18 years presenting to hospital with chest pain or dyspnea with a primary diagnosis of acute PTE were included. The diagnosis of acute PTE has been made using multidetector computed tomography (CT) angiography. CT angiography result was considered diagnostic for PTE in the presence of intraluminal filling defect or arterial cut-off. In patients who were contraindicated to undergo CT angiography because of a history of contrast allergy, patients with severe renal dysfunction (estimated glomerular filtration rates <30 mL/min) or pregnant women, the diagnosis of acute PTE was established using ventilation/perfusion (V/Q) scintigraphy and high probability scans were considered as diagnostic. Patients were excluded if they were admitted >24 hours after the symptom onset, if they had a prior history of PTE or an underlying disease limiting the life expectancy to less than one month (e.g. major trauma or high-grade cancer).

Demographic information, comorbidities, admission vital signs, echocardiographic findings, admission laboratory data before receiving treatment as well as main treatment protocol and survival status during hospitalization of included patients were recorded in prepared questionnaires by a trained physician.

We calculated simplified PESI score using variables of age >80 years, pulse rate ≥110 beats/minute, systolic blood pressure <100 mm Hg, history of cancer, chronic cardiopulmonary disease, and arterial oxygen saturation <90%.^3^

### Laboratory measurements

Venous blood samples on admission were obtained and collected in citrated tubes containing EDTA potassium salt additive as an anticoagulant. Blood samples were transferred to and analyzed by hospital laboratory within one hour. Total white blood cell, neutrophil, lymphocyte and platelet counts, hemoglobin, PDW and MPV were recorded for each patient. Biochemical analysis was performed to measure blood urea nitrogen (BUN), creatinine and blood glucose. For performing differential complete blood count analyses, an automated Coulter CBC H1 counter, which was calibrated on a daily basis was used. PLR was calculated as the platelet count divided by the absolute lymphocyte count per one cubic millimeter (mm^3^). NLR was calculated as the absolute neutrophil count divided by the absolute lymphocyte count per one cubic millimeter (mm^3^).

### Follow up

Patients were followed during the index hospital stay and regularly afterwards in the outpatient clinics for one year.

### Outcomes

The primary endpoint of interest in this study was in-hospital all-cause mortality. Major adverse cardiopulmonary events (MACPE), defined as a composite of the all-cause mortality and the need for thrombolysis, mechanical ventilation and surgical embolectomy during the index hospitalization and all-cause death during follow-up were secondary endpoints.

### Statistical analysis

Mean and standard deviation (SD) were presented for continuous variables and frequency (%) for categorical variables. For comparison between groups with and without clinical events, the independent *t* test, Mann-Whitney U rank sum test and the Pearson χ^2^ test, were used.

Receiver operating characteristics (ROC) curve was used to determine the area under the curve (AUC) and to identify the optimal cut-off point of MPV, PDW, PLR and the simplified PESI score in predicting different clinical outcomes including in-hospital death, in-hospital MACPE, and follow-up death. Pearson correlation test was done between MPV and total platelet counts, cardiac troponin levels, and systolic pulmonary arterial pressure as well as between platelet indices and the PESI risk score. All data analyses were performed using SPSS (Version 21.0; IBM SPSS Corporation, Chicago, IL) and *P* values <0.05 were considered statistically significant.

## Results

In this prospective cohort study, 492 patients with confirmed acute PTE were enrolled. The mean age was 62.1 ± 17.1 years and 48% of cases (236 patients) were male. Patient demographics, clinical presentations, and baseline laboratory and imaging findings are summarized in Tables 1-3.

**Table 1:** Patient characteristics in the study population and in groups with and without major adverse cardiopulmonary events (MACPE)

	**Total (n=492)**	**With MACPE (n=151)**	**Without MACPE (n=341)**	***P*** **value**
Age, year	62.1 ± 17.2	58.9 ± 18.1	63.5 ± 16.6	0.006
Male sex, n(%)	236 (48.0)	77 (51.0)	159 (46.6)	0.371
Seasonal distribution				
Spring	122 (24.8)	33 (21.9)	89 (26.2)	0.397
Summer	142 (28.9)	46 (30.5)	96 (28.2)	
Fall	111 (22.6)	40 (26.5)	71 (20.9)	
Winter	116 (23.6)	32 (21.2)	84 (24.7)	
Comorbidities				
Hypertension	206 (42.0)	55 (36.7)	151 (44.3)	0.115
Diabetes mellitus	72 (16.0)	24 (18.8)	48 (14.9)	0.309
Congestive heart failure	39 (7.9)	8 (5.3)	31 (9.1)	0.151
Hyperlipidemia on statins	50 (21.6)	15 (21.4)	35 (21.6)	0.976
COPD	43 (8.7)	9 (6.0)	34 (10.0)	0.146
Past/current smoker	63 (14.0)	22 (17.2)	41 (12.7)	0.215
Concurrent DVT	16 (5.2)	5 (5.7)	11 (5.0)	0.781
Active Cancer	34 (6.9)	10 (6.6)	24 (7.0)	0.867
Recent history of immobility	98 (19.9)	31 (20.5)	67 (19.6)	0.821
Recent long distance travelling	8 (1.6)	3 (2.0)	5 (1.5)	0.706
Oral contraceptives	20 (4.1)	4 (2.6)	16 (4.7)	0.290

COPD: chronic obstructive pulmonary disease.

**Table 2:** Clinical presentation and imaging findings in patients with and without major adverse cardiopulmonary events (MACPE)

	**Total (n=492)**	**With MACPE (n=151)**	**Without MACPE (n=341)**	**P value**
Hypothermia	131 (34.9)	38 (34.9)	93 (35.0)	0.985
Dyspnea	401 (81.5)	122 (80.8)	279 (81.8)	0.787
Tachypnea > 20 bpm	245 (57.0)	77 (60.2)	168 (55.6)	0.386
Pleuritic chest pain	68 (13.8)	12 (7.9)	56 (16.4)	0.012
Substernal chest pain	81 (16.5)	20 (13.2)	61 (17.9)	0.200
Syncope	55 (11.2)	31 (20.5)	24 (7.0)	<0.001
Tachycardia > 100 bpm	229 (47.6)	88 (60.3)	141 (42.1)	<0.001
RV enlargement	310 (64.2)	128 (85.3)	182 (54.7)	<0.001
RV diameter on CT (mm)	4.62 ± 0.94	4.46 ± 0.81	4.66 ± 0.98	0.471
RV dysfunction	288 (59.6)	123 (82.0)	165 (49.5)	<0.001
Right/Left ventricular ratio	0.79 ± 0.19	0.75 ± 0.14	0.81 ± 0.20	0.300
Systolic pulmonary pressure	50.6 ± 21.3	55.2 ± 20.0	48.0 ± 21.7	0.003
Clot on echocardiogram	33 (6.9)	27 (18.1)	6 (1.8)	<0.001
Presence of saddle embolus	51 (12.5)	32 (25.8)	19 (6.7)	<0.001
Segmental pulmonary emboli	119 (29.7)	19 (15.4)	100 (36.0)	<0.001

RV: right ventricle.

**Table 3:** Laboratory findings in patients with and without major adverse cardiopulmonary events (MACPE)

	**Total (n=492)**	**With MACPE (n=151)**	**Without MACPE (n=341)**	**P value**
Laboratory findings				
Hemoglobin (g/dL)	12.8 ± 2.4	12.8 ± 2.5	12.7 ± 2.3	0.607
White blood cell count (103 cells/nL)	11.3 ± 9.3	13.9 ± 15.5	10.2 ± 3.9	0.005
Neutrophil lymphocyte ratio	6.2 ± 6.2	6.8 ± 6.2	5.8 ± 6.2	0.206
Platelets (10^3^ counts/nL)	205.0 ± 84.9	183.6 ± 76.9	214.3 ± 86.6	<0.001
Mean platelet volume (femtoliter)	9.9 ± 1.0	10.1 ± 1.0	9.9 ± 1.0	0.019
Platelet distribution width	13.5 ± 6.1	13.6 ± 2.7	13.5 ± 7.0	0.914
Platelet lymphocyte ratio (PLR)	14.7 ± 14.5	15.7 ± 16.5	14.2 ± 13.5	0.338
Cardiac troponin I (ng/L)	0.45 ± 1.12	0.68 ± 1.40	0.35 ± 0.96	0.019
Serum d-dimer (µg/mL)	2.1 ± 2.0	2.4 ± 2.1	1.9 ± 1.9	0.178
Arterial partial pressure of O2 (mm Hg)	62.1 ± 28.2	64.4 ± 34.2	60.9 ± 24.6	0.337
Arterial partial pressure of CO2 (mm Hg)	37.7 ± 11.1	38.0 ± 11.7	37.5 ± 10.8	0.708
Blood glucose	138.7 ± 77.5	151.5 ± 93.5	133.6 ± 69.7	0.064
Serum creatinine (mg/dL)	1.3 ± 1.0	1.2 ± 0.5	1.3 ± 1.2	0.714
Alkaline phosphatase (IU/L)	262.0 ± 168.4	223.8 ± 104.9	277.7 ± 186.8	0.055
Alanine aminotransferase (IU/L)	66.4 ± 86.3	73.2 ± 76.1	63.4 ± 90.6	0.562
Aspartate aminotransferase (IU/L)	55.4 ± 75.7	55.0 ± 54.7	55.5 ± 83.5	0.974
Serum Total Cholesterol (mg/dL)	167.5 ± 46.1	161.3 ± 38.6	170.0 ± 48.7	0.095
Low density lipoprotein (mg/dL)	104.0 ± 37.2	96.9 ± 29.8	107.0 ± 39.6	0.034
Very low density lipoprotein (mg/dL)	31.6 ± 25.5	28.4 ± 10.4	33.2 ± 30.4	0.598

### Outcomes

Median length of stay (LOS) at hospital was 9 days (1^st^ and 3^rd^ quartiles 7, 12), and in-hospital death occurred in 42 (8.5%) patients. Ninety-seven patients (19.7%) received fibrinolytic therapy during the index hospitalization. Thirty-five (7.1%) and 23 (4.7%) patients underwent mechanical ventilation and embolectomy, respectively. In total, 151 patients (30.7%) experienced the composite endpoint of all-cause death, thrombolysis, mechanical ventilation and surgical embolectomy during the index hospitalization.

Patients were followed up for a median period of 13 months (interquartile range: 3-23 months). All-cause mortality occurred in 107 patients (21.7%) during the follow-up period.

### Platelet indices

Total platelet counts were lower in patients who suffered a major adverse event in hospital compared to others (183.6 ± 76.9 vs 214.3 ± 86.6 10^3^ cells/nL; *P* < 0.001). MPV was 9.9 ± 1.0 fl in the total cohort, and it was higher in those with MACPE as compared to their counterparts (10.1 ± 1.0 vs 9.9 ± 1.0; *P* =0.019).

PDW was 13.5 ± 6.1% in the total cohort and it was not different between groups with and without MACPE (13.6 ± 2.7% vs 13.5 ± 7.0%; *P* > 0.05). Similarly, PLR was 14.7 ± 14.5 in this study population and was not different between patients who had in-hospital MACPE compared to others (15.7 ± 16.5 vs 14.2 ± 13.5; *P* > 0.05).

**Figure 1:**
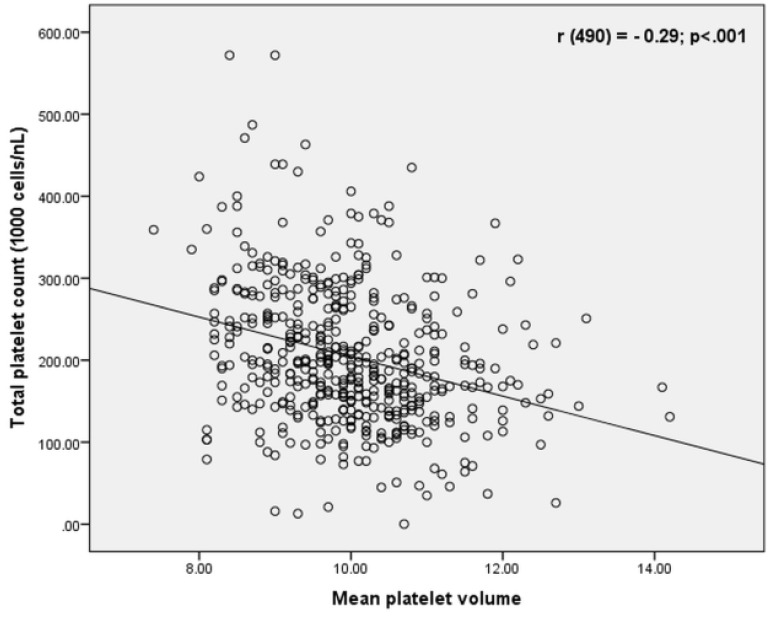
Correlation between MPV and total platelet count in this population.

Pearson correlation test revealed that MPV was inversely correlated with the total platelet count [*r*(490)= - 0.29; *P* < 0.001] (Figure 1). There was no correlation between MPV and cardiac troponin levels [*r*(401)=0.05; *P* =0.3]. Also there was no correlation between MPV and systolic pulmonary arterial pressure [*r*(343)=0.03, *P* = 0.4] in this study population. Unlike MPV (*r*=0.10, *P* = 0.14) and PDW (*r*=0.05, *P*= 0.51), PLR was correlated with the risk level determined by the simplified PESI score (*r*=0.25, *P*= 0.001).

### Prognostic performance

**Figure 2:**
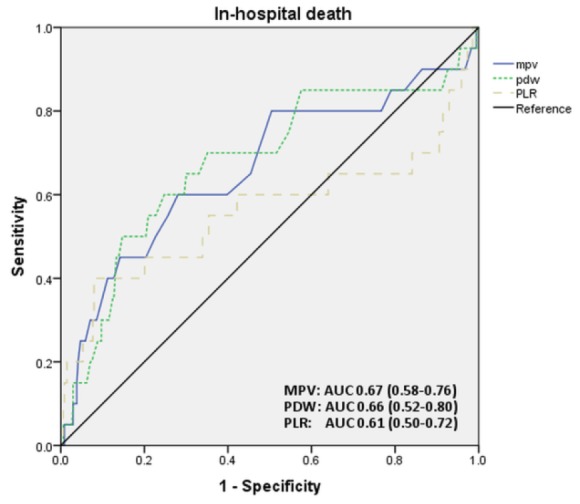
Receiver operating curve for evaluating sensitivity and specificity of platelet indices in predicting in-hospital death.

Figure 2 depicts the ROC curves of the predictive performance of different platelet parameters in predicting in-hospital mortality. The predictive performance of these indices in predicting various patient outcomes such as in-hospital mortality, in-hospital MACPE, and death during follow-up period are summarized in Table 4. MPV with a cut-off point of 9.85 fl has a sensitivity and specificity of 81% and 50%, respectively in predicting in-hospital mortality in patients with acute PTE, but it had lower performance in predicting composite endpoints (i.e. MACPE AUC 0.58; 95% CI 0.52-0.63) or long-term mortality (AUC 0.54; 95% CI 0.47-0.61).

**Table 4:** The predictive performance of mean platelet volume (MPV), platelet distribution width (PDW), and platelet-lymphocyte ratio (PLR), in prognosticating different clinical outcomes

	**AUC (95% CI)**	**Cut-point**	**Sensitivity**	**Specificity**
MPV				
In-hospital death	0.67 (0.58-0.76)	9.85 fl	81.0%	49.6 %
MACPE	0.58 (0.52-0.63)	9.85 fl	65.6%	52.5%
Follow up death	0.54 (0.47-0.61)	10.0 fl	51.4%	57.1%
PDW				
In-hospital death	0.66 (0.52-0.80)	13.60	66.7%	65.2%
MACPE	0.57 (0.51-0.64)	12.35	73.1%	46.7%
Follow up death	0.50 (0.42-0.59)	13.65	45.0%	66.4%
PLR				
In-hospital death	0.61 (0.50-0.72)	12.8	61.0%	63.2%
MACPE	0.47 (0.42-0.54)	9.8	52.1%	46.0%
Follow up death	0.65 (0.58-0.72)	11.4	63.1%	63.5%
Simplified PESI				
In-hospital death	0.80 (0.71-0.88)	1.5	77.3%	70.4%
MACPE	0.69 (0.62-0.77)	0.5	90.5%	42.1%
Follow up death	0.76 (0.66-0.86)	1.5	60.4%	80.5%

PDW with the cut-point of 13.6% and PLR with the cut-point of 12.8, respectively had an AUC of 0.66 (95% CI 0.52, 0.80) and 0.61 (95% CI 0.50, 0.72) in predicting in-hospital mortality. In general, platelet indices in our study showed poorer performance when compared to the well-established risk score of the simplified PESI (Table 4). In Table 5, clinical outcomes were compared between patients with different levels of MPV, PDW and PLR.

**Table 5:** Clinical outcomes in groups with different levels of mean platelet volume (MPV), platelet distribution width (PDW), and platelet-lymphocyte ratio (PLR)

	**MPV≤9.85 fl** **(n=231)**	**MPV>9.85 fl** **(n=261)**	***P***	**PDW≤13.6** **(n=241)**	**PDW>13.6** **(n=139)**	***P***	**PLR≤12.8** **(n=286)**	**PLR>12.8 ** **(n=182)**	***P***
SBP <90 mm Hg	13 (5.7)	26 (10.2)	0.18	18 (7.6)	11 (8.3)	0.76	16 (5.8)	20 (11.1)	<0.01
Cardiac arrest	2 (0.9)	2 (0.8)	1.00	2 (0.8)	2 (1.4)	0.62	2 (0.7)	2 (1.1)	0.64
Vasopressor treatment	3 (2.1)	18 (11.2)	<0.01	8 (5.3)	10 (11.8)	0.07	15 (8.1)	(5.9)	0.49
Oxygen saturation (%)	84.9 ±12.8	86.3 ±11.8	0.33	86.0 ±13.2	85.9 ±12.3	0.94	86.0 ±12.5	85.4 ±12.3	0.66
Blood urea nitrogen	21.5 ±11.9	26.3 ±17.8	0.04	22.1 ±13.3	26.3 ±18.6	0.16	23.3 ±14.4	25.1 ±17.3	0.47
Fibrinolytic therapy	38 (16.5)	59 (22.6)	0.08	45 (18.7)	21 (15.1)	0.37	58 (20.3)	34 (18.7)	0.67
Mechanical ventilation	5 (2.2)	30 (11.5)	<0.01	12 (5.0)	15 (10.8)	0.03	20 (7.0)	15 (8.2)	0.61
Surgical embolectomy	7 (3.0)	16 (6.1)	0.10	9 (3.7)	9 (6.5)	0.22	14 (4.9)	7 (3.8)	0.59
Hospital LOS (days)	9.5 ±4.9	11.0 ±9.2	0.02	10.3 ± 6.4	11.5 ±10.6	0.24	10.8 ±8.3	9.8 ± 6.5	0.18
In-hospital death	8 (3.5)	34 (13.0)	<0.01	7 (2.9)	14 (10.1)	<0.01	16 (5.6)	25 (13.7)	<0.01
MACPE	52 (22.5)	99 (37.5)	<0.01	63 (26.1)	45 (32.4)	0.19	87 (30.4)	57 (31.3)	0.83

LOS: length of stay, MACPE: major adverse cardiopulmonary events, SBP: Systolic blood pressure .

**Figure 3:**
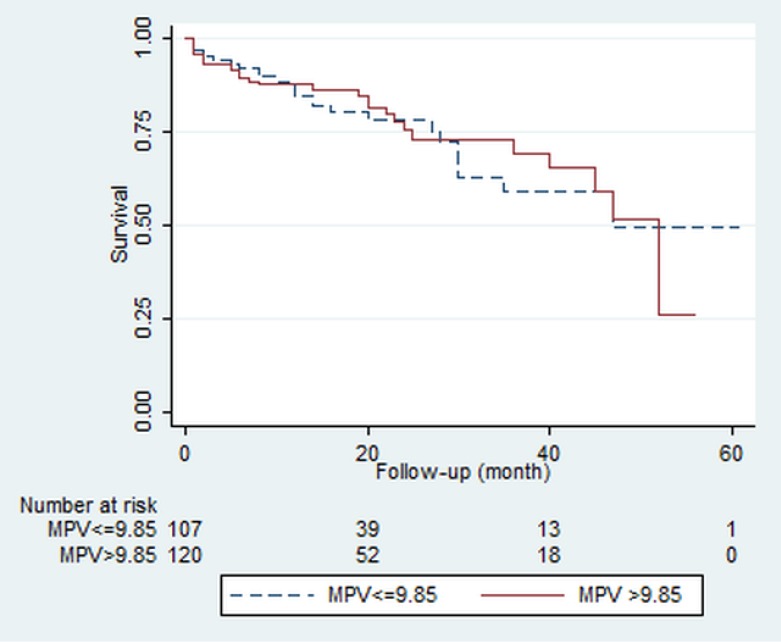
Patients’ survival during follow-up based on mean platelet volume at presentation.

We also performed a survival analysis to compare the all-cause mortality in the follow-up period between patients with different levels of MPV (Figure 3) and the Kaplan-Meier curve showed no difference between these two groups in terms of intermediate- to long-term mortality (log-rank test; *P* = 0.8).

## Discussion

In this study, we explored the prognostic value of platelet indices in predicting adverse outcomes in patients with acute PTE. Several key findings are noteworthy. MPV was higher in patients who had adverse events during their index hospital stay as compared to those who did not. All three indices had fair to good predictive performance in predicting in-hospital all-cause death. They performed poorer in predicting long-term mortality or in predicting a composite endpoint including death and other non-fatal adverse events. Despite having fair performance, established PTE risk scoring models such as PESI outperform these indices in predicting adverse outcomes.

Larger platelets have more glycoprotein Ib and glycoprotein IIb/IIIa receptors, and they release higher levels of thromboxane A2, platelet factor 4 and β-thromboglobulin,^5,7,12^ hence they are more metabolically and enzymatically reactive and have higher thrombotic potentials.^12^ Platelets are involved in the pathogenesis of various diseases and elevated MPV has been associated with higher risk of adverse events in several disease conditions, such as acute coronary syndrome, stroke.^13,14^ Higher MPV is also reported in the venous thromboembolism setting.^15,16^ It has been reported to be a risk indicator for predicting the possibility of PTE in patients with unprovoked DVT.^16,17^

Considering the presence of hypercoagulable state in venous thromboembolism and acute PTE and purported pro-thrombotic properties of platelets with higher MPV, it is expected to see a correlation between high MPV and clinical outcomes in this specific disease. Several studies have explored and demonstrated its diagnostic value in diagnosing acute PTE among the suspected patients.^18^ Our findings were in line with the findings of Kostrubiec et al which reported MPV to be an independent predictor of mortality in patients with acute PTE.^19^ A study by Günay et al showed that MPV and PDW can be useful in determining the severity of pulmonary vascular bed obstruction in PTE (i.e. massive vs submassive).^20^ Several studies suggested its correlation with right ventricular (RV) dimensions and the severity of RV dysfunction.^15,21,22^ Nevertheless, these studies were not consistent so far and some studies failed to find a correlation between MPV and disease severity.^21,23^

In this study, the simplified PESI outperformed the platelet indices in prognosticating patients with acute PTE. Akgülü et al in a study showed that the predictive performance of extensively-studied risk scores such as the simplified PESI can be improved by addition of factors such as MPV.^24^

Like several previous studies, MPV correlated inversely with the total platelet count. This can be attributed to the consumption of platelets for thrombus formation and a compensatory production of larger platelets from megakaryocytes in the bone marrow.^22,25^

Previous studies have showed a relationship between PLR and a high risk of in-hospital mortality in patients with acute PTE and found it to be correlated with established risk scores (e.g. simplified PESI).^23,26^ Similarly, in this study, PLR was correlated with the simplified PESI, whilst the other two indices lacked such correlation. However, the PLR levels were not different between patient with and without adverse events and it only had a fair predictive performance in predicting in-hospital mortality.

It is important to delineate the pathophysiologic mechanisms with which the activated platelet is linked to acute PTE itself and moreover to the adverse events in patients with acute PTE. A vicious cycle between the platelet activation and pulmonary vascular bed obstruction is the most legitimate explanation. The mechanisms that contribute include sympathetic activation resulted from hypoxemia^15,27^ which in turn leads to platelet activation and the release of angiotensin II, pulmonary vasoconstriction due to the release of thromboxane A2 from platelets,^6,28,29^ hypercoagulability secondary to an increase in the plasma levels of platelet factor-4 and β-thromboglobulin^29^ and increase in platelet-fibrinogen binding resulted from the overexpression of adhesion molecules such as P-selectin, glycoprotein Ib and glycoprotein IIb/IIIa in platelets.^5,30^

There are several potential limitations to this study. This study was a single-center observational study and it was subject to biases that are innate to observational studies. Our study included patients of a single center and one snap shot of blood samples has been utilized at the time of admission. Serial measurement and study of temporal changes were not possible in our study setting. There may be also racial and ethnical differences in normal ranges in a given population.

## Conclusion

In conclusion, platelet indices had only fair-to-good predictive performance in predicting in-hospital all-cause death. Despite having fair prognostication, established PTE risk scoring models such as simplified PESI outperform these indices in predicting adverse outcomes.

## Competing interests

The authors declare that there are not conflicts of interest.

## Ethical approval

The study was approved by the Ethics committee of Tabriz University of Medical Sciences and informed consent was obtained from all participants prior to study enrollment.
